# Interventional Feasibility Study Investigating Virtual Reality‐Based Gait Assessment With Orthoses in People With Multiple Sclerosis: A Study Protocol

**DOI:** 10.1111/cts.70728

**Published:** 2026-09-09

**Authors:** Kevin Marcaccini, Alessandro Aleo, Stefania Pozzi, Giorgia Sgambato, Lorenzo Chiari, Maria Giovanna Verrengia, Domenica Chiara Milano, Serena Moscato, Sabato Mellone, Valerio Antonio Arcobelli, Loredana Sabattini, Silvia Orlandi

**Affiliations:** ^1^ Department of Electrical, Electronic, and Information Engineering “Guglielmo Marconi” (DEI) University of Bologna Bologna Italy; ^2^ Rehabilitation Bioengineering Lab IRCCS Istituto Delle Scienze Neurologiche di Bologna Bologna Italy; ^3^ IRCCS Istituto Delle Scienze Neurologiche di Bologna Bologna Italy; ^4^ Department of Biomedical and Neuromotor Sciences (DIBINEM) Alma Mater Studiorum ‐ University of Bologna Bologna Italy; ^5^ UO DATER Riabilitazione Ospedaliera Azienda USL Bologna Bologna Italy; ^6^ Health Sciences and Technologies, Interdepartmental Center for Industrial Research (CIRI‐SDV) University of Bologna Bologna Italy

**Keywords:** gait impairments, inertial sensors, multiple sclerosis, neurorehabilitation, physiological signals, sensor‐based monitoring, virtual reality, walking braces, wearable sensor

## Abstract

Multiple sclerosis (MS) often leads to significant impairments in gait and mobility. Although walking braces are commonly prescribed, their real‐world effectiveness is limited by insufficient adaptation. Traditional clinical assessments rely on subjective scales and may not fully reflect daily‐life challenges. Immersive virtual reality (VR) and wearable sensors offer promising solutions for ecological monitoring, but their combined use remains unvalidated for orthotic support in MS. This interventional study examines the feasibility of a novel, VR‐based framework designed to support gait assessment with a passive walking brace. Twelve participants with MS will undergo a protocol combining four ambulatory sessions and a week‐long home‐monitoring phase. Standardized gait tests will be performed with and without the brace across both simulated scenarios and real‐world conditions. Multimodal data collected via inertial and physiological wearable sensors will characterize spatiotemporal gait parameters, balance, posture, and physiological responses, alongside standardized clinical scales and subjective questionnaires. Overall feasibility will be evaluated directly through user usability and acceptability, recruitment and retention rates, safety, protocol adherence, and data completeness. Given that this pilot study is not powered to assess clinical efficacy, statistical analysis will focus strictly on descriptive and exploratory comparisons across conditions and sessions. This translational framework aims to establish robust feasibility metrics to support the design of future, larger‐scale controlled trials. Ultimately, these preliminary observations will inform more ecologically valid, personalized rehabilitation strategies to improve the integration of assistive technologies into patients' daily lives.

**Trial Registration:** ClinicalTrials.gov, NCT07096700. Registered July 24, 2025

## Study Highlights

**What is the Current Knowledge on the Topic?:**

Gait impairment significantly impacts the quality of life in people with multiple sclerosis. Although passive walking braces are widely used to assist ambulation, limited ecological training and subjective clinical tools constrain their real‐world adaptation and adaptation monitoring. Virtual reality and wearable sensors offer promising solutions for objective evaluation, but their combined application remains underexplored.

**What Question Did This Study Address?:**

This study protocol outlines a novel methodological framework to evaluate the technical feasibility, safety, usability, and user acceptability of an integrated, VR‐based monitoring system designed to test gait performance with a passive walking brace in VR environments that closely reproduce real‐life conditions.

**What Does This Study Add to Our Knowledge?:**

This protocol presents a unique, two‐phase design that pairs standardized ambulatory tasks in multi‐scenario virtual environments with continuous, home‐based ecological monitoring. It provides a methodology to gather descriptive spatiotemporal, physiological, and digital diary data from a small cohort.

**How Might This Change Clinical Pharmacology or Translational Science?:**

By validating a multidimensional feasibility framework and establishing baseline compliance metrics, this study will support the robust design of subsequent, larger‐scale controlled trials. In translational science, it establishes an objective, digital‐health‐supported pathway to support neurorehabilitation protocols and personalize the evaluation of assistive devices.

## 1 Introduction

Multiple sclerosis (MS) is a chronic autoimmune neurodegenerative disease affecting the central nervous system, characterized by a wide range of symptoms. In Italy, approximately 126,000 individuals are affected [1], with a higher prevalence in women (3:1), likely due to hormonal and genetic factors [1]. Gait impairment is among the most common manifestations in MS and represents a major focus of rehabilitation, including the use of orthotic devices to support walking [2, 3]. Rehabilitation aims to enhance motor coordination, posture, balance, flexibility, and muscle strength, ultimately improving overall mobility [4].

Gait analysis plays a key role in clinical assessment, offering insights into motor function, coordination, and balance through kinematic, dynamic, and electromyographic (EMG) evaluations [5]. Current gait rehabilitation strategies in MS mainly rely on conventional physiotherapy and orthotic training, which require time and specific effort, particularly for postural and ambulatory support [6]. A promising device strategy involves the use of a wearable passive orthosis designed to enhance hip flexor propulsion and trunk stability, thereby improving gait efficiency, balance, and walking distance [7].

Wearable sensors have been increasingly used to assess gait in MS [8]. Studies have shown how inertial sensors provide reliable measures of gait phases, stride length, and symmetry, as well as fatigue and postural stability [9, 10]. These measurements offer valuable insights into motor function and can help monitor rehabilitation in real‐life settings.

Machine learning (ML) techniques represent an innovative, promising instrument in gait analysis, automating kinematic assessments through inertial data. For instance, Marimon et al. [11] achieved 92.4% accuracy in extracting gait parameters, generating data useful for diagnostic and preventive aims, while Greve et al. [12] were able to automatically segment gait phases with an average error of 3.4%.

Discomfort and stress are major barriers to the adoption of assistive devices in gait rehabilitation [13], often leading to their abandonment. Usually, their quantification is done by self‐reported questionnaires, but they lack objectivity [14]. Recent research has used ML and deep learning on physiological signals to objectively quantify stress and comfort levels. For example, Albertetti et al. [15] analyzed electro‐dermal activity (EDA) and blood volume pulse (BVP) signals, obtaining an F1‐score of 0.71. Hota and Park [16] reported > 98% accuracy using variables related to respiration, sweat, and heart rate (HR). Camardella et al. [17] further analyzed the relationship between perceived comfort and physiological parameters (EDA, HR, and HR variability, HRV) in exoskeleton use.

Virtual Reality (VR) has recently emerged as a promising tool to enhance motor function and improve quality of life in people with MS [18]. VR refers to technologies that simulate interactive, computer‐generated environments, enabling users to experience and interact with realistic scenarios [19]. Fully‐immersive VR, for instance, relies on head‐mounted displays that create a strong sense of presence within the virtual space. In contrast, augmented or mixed reality (AR or MR) integrates selected elements of the physical world into predominantly virtual settings, allowing real‐time interaction between digital and real components. Mixed reality, in particular, can support users who benefit from maintaining physical‐world references while interacting with virtual environments, supporting users' sensory perception and motor control [20] or mitigating VR‐related side effects such as cybersickness [21]. Cybersickness, specifically, is a common condition, similar to motion sickness that occurs in VR due to a mismatch between visual input and bodily sensory feedback. This type of technology may be particularly valuable for individuals with MS, as many demonstrate good performance during standardized clinical assessments but encounter significant difficulties when using assistive devices in daily life [6, 22]. VR has the potential to bridge this gap by providing realistic and safe environments for evaluation and guided task exposure within clinical settings [23, 24, 25]. Nevertheless, its application to gait assessment and orthotic familiarization, especially in people with MS, remains largely underexplored in the literature [26]. The VIrtual Reality for gait Training in mUltiple sclErosis (VIRTUE) project seeks to address this gap by leveraging VR to replicate everyday walking challenges in people with MS using passive orthoses.

### 1.1 Study Objectives and Overview

This study explores the feasibility of implementing a monitoring system for gait characterization with a passive orthosis in virtual reality environments simulating real‐life conditions. By integrating wearable sensor data with standard clinical functional scales, we aimed to determine whether this approach provides clinicians with objective insights into patient adaptation and gait trends. Outcomes such as energy expenditure, perceived fatigue, and cognitive load will be quantified through both standardized questionnaires and sensor‐derived gait and physiological measures.

The project will be implemented in two phases. An ambulatory phase will include the repetition of standardized gait tests performed with the passive orthosis supported by VR and wearable inertial sensors in simulated environments. A home‐based phase will focus on the feasibility of ecological monitoring of gait with the passive orthosis and wearable sensors during activities of daily living. The proposed system, which integrates VR and wearable technologies, will be examined in terms of feasibility, usability, and acceptability, while gait‐related data will be collected for purely descriptive purposes to examine variability across conditions and sessions. Spatiotemporal gait parameters, routinely used in clinical practice and captured via inertial sensors, will be collected both during VR sessions and in real‐life contexts to monitor descriptive changes in gait patterns while using the orthosis.

## 2 Methods

### 2.1 Study Design and Participants

This is a single‐center interventional feasibility study evaluating the feasibility, safety, acceptability, and usability of a VR‐based gait assessment and orthotic familiarization framework combined with passive orthosis use in people with multiple sclerosis. The study is investigator‐initiated and nonprofit funded. It will last 1 year and will be conducted in accordance with the ethical principles established in the Declaration of Helsinki and has been approved by the local Ethical Committee (approval no. 29‐2025‐DISP‐AUSLBO). Any substantial modifications to the study protocol will be submitted to the Ethics Committee as amendments and implemented only after approval. This is the first protocol version (001.2024.ISNB.UOSI‐RIAB‐MS) and has been registered on clinicaltrials.gov (registration ID: NCT07096700).

The project will involve 12 patients with MS, aged between 18 and 65 years, referred to the UOSI Riabilitazione Sclerosi Multipla (UOSI‐RSM) of the IRCSS Istituto delle Scienze Neurologiche di Bologna (IRCCS‐ISNB), who meet the inclusion and exclusion criteria detailed in Table 1.

**TABLE 1:** Inclusion and exclusion criteria.

Inclusion criteria	Exclusion criteria
Confirmed diagnosis of MS. Age between 18 and 65 years. Both sexes. Brief International Cognitive Assessment for Multiple Sclerosis (BICAMS) > 100. Expanded Disability Status Scale (EDSS) score between 3 and 6. Berg Balance Scale (BBS ≥ 46). Clinical indication for the use of the ExoBand orthosis for gait assistance. Modified Fatigue Impact Scale (MFIS), physical component ≤ 20. Signed informed consent.	Presence of severe or ongoing visual or auditory deficits. Relapse within the last 3 months. Presence of severe anxiety or depression. Severe spasticity patterns in the lower limbs or fixed distal tendon retractions. Presence of VR‐induced motion sickness symptoms.

Prior to enrollment, the principal investigator (PI) will verify that each potential participant meets the study eligibility criteria. Once confirmed, the PI or a designated team member will provide a detailed explanation of the study objectives, procedures, and potential risks and benefits during an interview, preferably conducted in the presence of a caregiver. Only after confirming that the participant has fully understood the information will written informed consent be obtained before initiating any study‐related procedures (see Supporting Information).

### 2.2 Study Objectives

The primary objective of this study is to investigate the feasibility of a monitoring system designed to test gait performance with a passive walking brace in VR environments that closely reproduce real‐life conditions, specifically assessing the framework's usability and overall acceptability. The system integrates wearable inertial sensors with routinely used functional clinical scales with the goal of determining whether this technological approach can provide clinicians with additional objective information, particularly regarding patients' adaptation to the orthosis and trends in gait parameters.

The secondary objectives include assessing the role of VR in replicating everyday contexts, such as the home, workplace, and public spaces, and examining its potential to enhance adaptation to assistive devices in daily life. The study also aimed to examine user satisfaction and technology acceptance in people with MS through validated questionnaires. Furthermore, we will analyze the feasibility of repeated longitudinal assessments and preliminary trends in gait and balance outcomes over time through a follow‐up session carried out 2 months after the last usage of the proposed system. Another objective is to understand the feasibility of characterizing orthosis‐assisted gait in individuals with MS using data collected from wearable inertial sensors and from video recordings processed through skeletal motion models during ambulatory sessions. Finally, the study will determine the technical feasibility of establishing preliminary, proof‐of‐concept ML pipelines. These exploratory analyses are strictly aimed at evaluating data quality, feature extraction workflows, and pipeline viability, rather than developing generalizable predictive models.

This study is designed as an interventional feasibility study and is not powered to detect clinical efficacy outcomes. Clinical, gait, and physiological measures will therefore be interpreted descriptively and exploratorily.

### 2.3 Study Materials

The study makes use of a range of devices and validated instruments to support the implementation of the system and the collection of gait, physiological, and user interaction data within the VR system. Virtual environments will be delivered through the Meta Quest 3 (Meta Platforms Inc.), a commercially available headset that provides immersive and mixed reality scenarios simulating daily‐life contexts. Lower limb movements are tracked using the Meta Quest 3 embedded camera‐based tracking system, which allows real‐time avatar representation and enhanced visual feedback during gait. Real‐time gait analysis is performed with the mTest3 system (mHealth Technologies), a certified medical device connected to a smartphone application that estimates spatiotemporal gait parameters such as step length, cadence, walking speed, and gait phase duration through two inertial measurement units (IMUs) positioned on the shoelaces and three additional IMU sensors attached to the torso and shoulders. Physiological responses will be monitored with the Embrace Plus (Empatica Inc.), a CE‐marked wristband capable of recording photoplethysmography (PPG), electrodermal activity (EDA), skin temperature, and acceleration, thus allowing the analysis of HRV as well as stress and arousal levels during VR exposure and brace use [27, 28, 29]. Two GoPro HERO12 cameras will be used to capture gait from multiple perspectives and enable markerless motion capture using skeleton‐based models. High‐performance gaming laptops support the VR applications and data acquisition, while a secondary laptop will be dedicated to questionnaire administration and secure data storage. The orthosis used in the study is the ExoBand (Moveo s.r.l.), a passive, textile‐based hip flexion‐assist brace registered as a medical device, which uses elastic tension to facilitate leg swing and aims to reduce fatigue and improve stability both in clinical and daily‐life settings.

In addition to these devices, a set of validated questionnaires and clinical scales will be administered to support the multidimensional characterization of participants and to investigate the feasibility of collecting data across multiple domains. Cognitive and physical functioning, fatigue, and walking ability will be quantified using the Brief International Cognitive Assessment for MS (BICAMS) [30], the Modified Fatigue Impact Scale (MFIS) [31], and the 12‐item MS Walking Scale (MSWS‐12) [32, 33]. Quality of life will be assessed with the Multiple Sclerosis International Quality of Life questionnaire (MusiQoL) [34], while balance and mobility data will be collected using the Berg Balance Scale (BBS) [35], the Expanded Disability Status Scale (EDSS) [36], the Timed 25‐Foot Walk Test (T25FWT) [37], the 2‐Minute Walk Test (2MWT) [38], and the Timed Up and Go test (TUG) [39]. Workload and VR‐related experiences will be evaluated with the NASA Task Load Index (NASA‐TLX) [40], the Simulator Sickness Questionnaire (SSQ) [41], and the iGroup Presence Questionnaire (IPQ) [42]. Finally, technology acceptance and user satisfaction will be assessed in terms of feasibility and acceptability using the Technology Acceptance Model (TAM) [43] and the Tele‐healthcare Satisfaction Questionnaire—Wearable Technology (TSQ‐WT) [44].

As part of this project, a dedicated software application named VIRTUE4ms has been developed to simulate real‐life scenarios while administering standardized gait tests. The simulator incorporates a task‐specific gamification framework designed to enhance user engagement and adherence. It has been tailored for the 25FWT and TUG tests, integrating automated logic for both test administration and result recording. Moreover, the simulator includes a dedicated module to monitor the occurrence of potential VR‐related side effects, such as cybersickness. In case symptoms of cybersickness are detected or reported by a participant, an MR version of the software will be employed to mitigate these effects.

### 2.4 Study Procedure

The intervention will consist of five sessions. All the ambulatory sessions will be held in the UOSI‐RSM IRCCS ISNB.

In the preliminary session (T0), eligible participants will be recruited, and the Case Report Form (CRF) will be completed to document clinical and functional information. Participants will then undergo a short VR experience to screen for potential side effects such as cybersickness. If symptoms occur, subsequent sessions will be conducted in augmented reality rather than fully immersive VR. To quantify adverse effects, participants will complete the SSQ and will wear the EmbracePlus wristband throughout all sessions. The TAM questionnaire will also be administered. Baseline measures will include the BICAMS for cognition, the MFIS for fatigue, and the EDSS for disease progression.

Once all 12 participants are enrolled and have completed session T0, the first ambulatory session (T1) will take place. Participants will perform two standardized gait and balance tests in a clinical corridor (i.e., T25FWT and TUG) under four conditions: (i) without ExoBand or VR; (ii) with VR only; (iii) with ExoBand only, and (iv) with both VR and ExoBand. In VR conditions, tests will be conducted within fully immersive environments simulating real‐life contexts (e.g., museum corridor, supermarket aisle, basketball court, park, sidewalk, house corridor). For participants reporting cybersickness at T0, augmented VR will be used instead. If possible, the same virtual environment will be maintained across sessions for consistency, selected based on participant preference or clinician recommendation. Participants will then perform a final gait test, the 2MWT, both with and without the Exoband. The 2MWT will not be conducted in a virtual environment, as the test requires participants to walk over a long distance (20 m) for a prolonged duration (2 min), which is not feasible in VR due to the need for the headset to be connected to a PC via cable. In all conditions, rest periods will be provided between tests, or upon participant request, to prevent fatigue. During each test, physiological parameters will be collected with the EmbracePlus wristband. Spatiotemporal gait data, including step length, stance/swing duration, and walking speed, will be collected using the mTest3 system with inertial sensors attached to the shoe laces and trunk. In VR conditions, the Meta Quest 3 cameras will be used to track participants' body movements and replicate them on a virtual avatar. All trials will also be video recorded with the two GoPro cameras for markerless motion analysis. Before and after each VR and brace condition, the NASA‐TLX questionnaire will be administered to quantify perceived physical and mental workload. At the end of T1, participants will complete the SSQ and the IPQ to record the occurrence of motion sickness and the sense of presence. Additional measurements will include the MusiQoL, BICAMS, MFIS, BBS, and the MSWS‐12. Given the limited sample size (*n* = 12), it will not be possible to divide participants into two separate groups (e.g., VR vs. non‐VR, or Exoband vs. no Exoband). Therefore, a within‐subject design will be adopted, with each participant performing all experimental conditions. To minimize potential carry‐over effects associated with the use of the passive orthosis, conditions involving the walking brace will be performed after those without it. The order of the VR condition (with or without Exoband) relative to the non‐VR condition will follow a simple allocation sequence generated before enrollment, so that half of the participants (*n* = 6) perform the VR condition first and the other half perform the non‐VR condition first.

For the following 7 days (Th), home‐based monitoring will be conducted to assess the feasibility of data collection in real‐life conditions, with participants asked to wear the ExoBand brace, mTest3 inertial sensors, and the EmbracePlus wristband for at least 8 h per day. Adherence to device usage and data completeness will be monitored throughout this period. Participants will be asked to keep a digital diary documenting daily activities, sleep–wake cycles, and medication use to contextualize the collected data and explore the feasibility of self‐reported logging.

On day 8, participants will return for session T2, replicating all T1 activities. In addition, they will complete the TSQ‐WT questionnaire to quantify participant satisfaction with wearable devices and the TAM questionnaire to assess technology acceptance after home use.

Two months later, participants will attend a follow‐up session (T3) in which T1 activities will be repeated without VR. This final session will examine the feasibility of repeated measurements over time and provide preliminary observations on potential changes in gait or other outcomes since earlier sessions.

**FIGURE 1:**
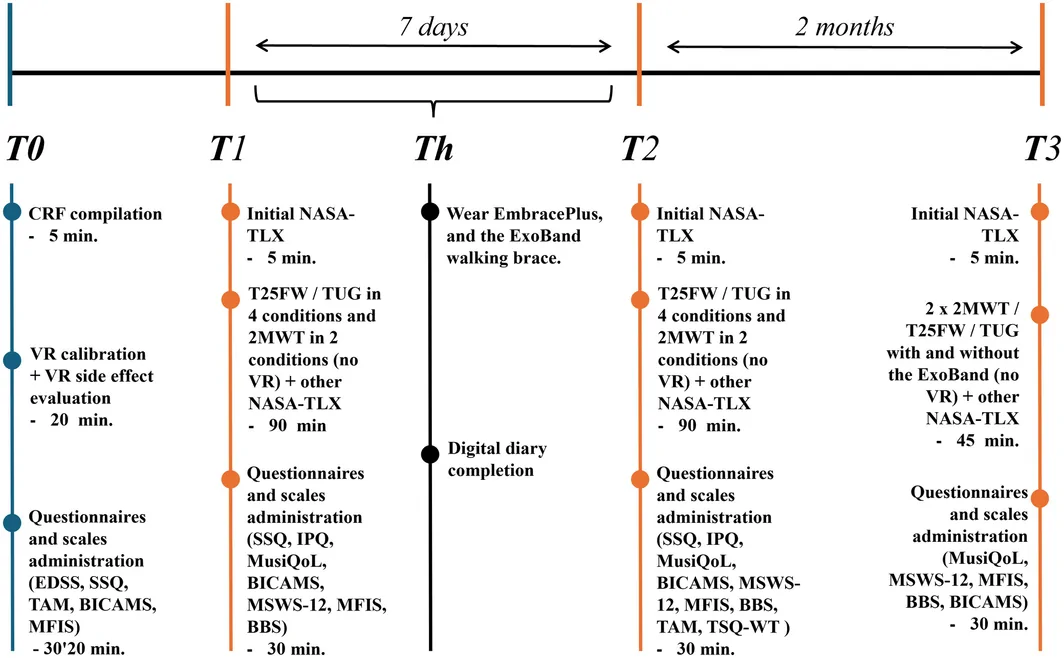
Intervention summary. 2MWT, 2‐minute walk test; BBS, Berg balance scale; BICAMS, Brief International Cognitive Assessment for Multiple Sclerosis; CRF, clinical record form; EDSS, Expanded Disability Status Scale; IPQ, Igroup presence questionnaire; MFIS, Modified Fatigue Impact Scale; MSAQ, motion sickness assessment questionnaire; MSWS‐12, twelve item MS walking scale; MusiQoL, Multiple Sclerosis International Quality of Life; NASA‐TLX, National Aeronautic and Space Administration task load index; T25FWT, timed 25‐foot walk test; TAM, technology acceptance model; TSQ‐WT, tele‐healthcare satisfaction questionnaire—wearable technology; TUG, timed up and go; VR, virtual reality.

Figure 1 presents a detailed overview of the intervention and the planned duration of each session.

### 2.5 Data Collection

Throughout all the sessions, trial conduct will be monitored continuously by the study investigators. Monitoring procedures will include verification of protocol adherence, completeness of data collection, and reporting of adverse events at each study session. Given the small sample size, short duration, and minimal‐risk nature of the study, no independent or external trial monitoring is planned.

For each participant, demographic and clinical data will be collected together with scores from the administered questionnaires. Physiological signals will be acquired using the EmbracePlus wearable device, including PPG, which records variations in blood volume associated with each heartbeat, enabling estimation of heart rate and analysis of HRV; EDA, which reflects sympathetic activation through eccrine sweat gland activity and serves as an indicator of physiological arousal, with pain‐related parameters extractable from the raw signal as well as from its tonic (slow changes) and phasic (rapid changes) components [8]; skin temperature (SKT), providing an indirect measure of sympathetic nervous system activity primarily influenced by peripheral blood flow; and acceleration, capturing physical activity and movement‐related data.

These signals will be analyzed to quantify stress levels, physical and mental workload associated with VR use, and potential side effects during task execution. In addition, accelerometric data from the mTest3 system will be collected to capture lower limb and trunk motion, allowing the derivation of objective gait parameters and the characterization of gait across sessions. Video recordings of gait tests will be processed through markerless skeleton‐based motion analysis to extract spatiotemporal gait features. This approach will be tested through a comparison with wearable sensors, given its reduced impact on natural gait [45]. Both physiological and motion‐related data will be considered alongside questionnaire outcomes to examine patterns in user experience and gait characteristics.

### 2.6 Endpoints and Statistical Analysis

The feasibility sample size of 12 participants with MS was chosen to provide a sufficient dataset to analyze the practicality of implementing a VR‐based intervention combined with a passive orthosis. Accounting for an estimated 20% dropout, the sample size is consistent with methodological recommendations for feasibility studies [46].

#### 2.6.1 Primary Endpoint

Feasibility of integrating VR and wearable sensors during gait with the passive orthosis in MS. Feasibility will be assessed based on participant adherence to the protocol, completeness of data collection, safety, and overall acceptability of the intervention.

#### 2.6.2 Secondary Endpoints

- Descriptive changes and trends in gait and balance parameters across sessions (T1, T2, and follow‐up T3), based on spatiotemporal parameters and clinical test results (2MWT, T25FWT, TUG).
- Associations between physiological signals, wearable/video‐based kinematic data, and subjective clinical or workload scores.
- Feasibility of establishing preliminary, proof‐of‐concept ML pipelines for gait [47, 48, 49] and physiological data, focusing on technical performance, data quality, and evaluating pipeline viability to inform future predictive models in adequately powered studies.

Continuous data (e.g., gait parameters, questionnaire scores) will be summarized using descriptive statistics (means, medians, standard deviations). Comparisons between sessions (T1–T2 and T1–T2–T3) will be conducted using paired or nonparametric tests to examine trends and variability in the data. Correlations between clinical scores and sensor/video‐based measures will be determined with Pearson or Spearman coefficients. Associations between physiological signals and subjective workload/motion sickness (NASA‐TLX, IPQ, and SSQ) will also be investigated through correlation analyses. The feasibility of establishing preliminary ML pipelines (e.g., using simpler algorithms like regularized linear models or support vector machines) to explore associations with gait quality and participant well‐being from inertial and physiological data will be evaluated. Although the study includes only 12 participants, each participant is expected to contribute multiple gait trials, with each gait cycle constituting an individual analysis instance for feature extraction and model input. Because these instances are not statistically independent, model evaluation will be performed leave‐one‐subject‐out cross‐validation (LOSO‐CV), ensuring that all gait cycles from a given participant are held out together during testing to prevent data leakage, mitigate potential model overfitting and overly optimistic performance estimates. Given the pilot nature of the study, all ML analyses will remain strictly exploratory and results will be reported using descriptive performance metrics (e.g., balanced accuracy, F1‐score) to demonstrate pipeline feasibility rather than model generalizability.

### 2.7 Data Management

The PI will act as data controller. Each participant will be assigned a pseudonymization code. Paper‐based records (i.e., patient record forms) will be stored at the UOSI‐RSM, IRCCS ISNB, and will be the only documents containing identifiable information. The master file linking pseudonymization codes with personal data will be securely retained by the PI.

For each participant:

- Questionnaires and scale data from clinical sessions will be entered directly into REDCap (electronic data capture system) and recorded in the electronic CRF.
- Home‐based data will be collected via Microsoft Forms, pseudonymized prior to storage, and then archived in REDCap and the CRF.
- Physiological, spatiotemporal, and VIRTUE4ms‐extracted data will be pseudonymized and analyzed with dedicated in‐home programming codes.

All data will be handled in compliance with the General Data Protection Regulation (GDPR) and retained for 10 years. Pseudonymized datasets will be stored in password‐protected systems (i.e., online platforms such as REDCap or protected servers) accessible only to authorized research staff. Data analysis will be performed using statistical software tools such as Stata SE 16.0 or MATLAB.

Missing data will be reported and described for each outcome measure. Given the exploratory nature and small sample size of this pilot study, no formal imputation procedures will be applied. Analyses will be conducted on available data only.

### 2.8 Ethics and Dissemination

The study will be conducted in accordance with the ethical principles outlined in the Declaration of Helsinki and has received prior approval from the local Ethics Committee (approval no. 29‐2025‐DISP‐AUSLBO). Any future amendments to the study protocol will be submitted to the Ethics Committee for review and approval as an amendment. Participation will be entirely voluntary. Informed consent for study participation and authorization for the processing of personal data will be obtained from all participants using dedicated consent forms.

The research team is committed to the timely and comprehensive dissemination of the study findings. Results will be presented at scientific conferences and submitted for publication in peer‐reviewed international journals.

The funders provide financial support for the development and implementation of the project but have no role in the scientific or operational conduct of the trial. Specifically, they are not involved in study design or protocol development, trial conduct or site management, nor in data collection, management, analysis, or interpretation. They have no role in manuscript drafting, review, or approval. All scientific, methodological, and dissemination decisions rest solely with the Principal Investigator and the academic research team. No mechanisms to mitigate funder influence were required, as the funders have no direct involvement in trial governance or data analysis.

### 2.9 Patient and Public Involvement

Patients and the public were not formally involved in the design or conduct of this study. However, the research objectives will be informed by clinical experience and patient needs routinely observed in rehabilitation practice. Study results will be shared with participants and relevant patient communities.

## 3 Results

### 3.1 Trial Status

The study protocol was approved by the local Ethics Committee (approval no. 29‐2025‐DISP‐AUSLBO) and registered on ClinicalTrials.gov (NCT07096700). Participant recruitment and experimental procedures commenced in April 2026 and are currently ongoing. Prior to patient enrollment, preliminary technical testing and pilot evaluations of the VIRTUE4ms software application, Meta Quest 3 integration, and sensor synchronization protocols were successfully conducted on neurotypical individuals to ensure system stability, safety, and data logging reliability.

### 3.2 Expected Outcomes

As an interventional feasibility study, primary results will quantify protocol adherence, recruitment and retention rates, safety, and technology acceptability across the four experimental ambulatory conditions and the 7‐day home‐monitoring phase. Secondary results will yield descriptive characterizations of spatiotemporal gait parameters, physiological arousal markers (EDA, HRV, skin temperature), and markerless video kinematics. Machine learning pipelines will be evaluated as proof‐of‐concept workflows to assess feature extraction viability and technical performance. Complete data analysis and endpoint evaluations will be reported upon trial completion.

## 4 Discussion

In current clinical practice, the adaptability and effectiveness of walking aids in people with MS are typically assessed in controlled, non‐ecological environments using standardized scales and subjective questionnaires. Such approaches often fail to capture the challenges patients face in daily life, potentially leading to an overestimation of their functional capabilities. The VIRTUE4ms system seeks to address this gap by testing the feasibility of a more naturalistic yet ambulatory evaluation of gait performance. Through immersive, personalized virtual environments, researchers can replicate real‐world contexts while still operating in a clinical setting. Wearable sensors and markerless skeleton‐based video analysis enable objective and continuous gait monitoring. Additionally, exploratory ML techniques are evaluated to test the feasibility of automated feature extraction from complex multimodal data, laying the groundwork for data analysis in future larger trials.

This study aimed to investigate the feasibility and added value of using VR and wearable technologies to facilitate functional adaptation to walking aids in both clinical and home‐based settings. While several studies have used VR in MS rehabilitation [50, 51], the majority of gait‐related interventions rely on treadmill‐based or robotic systems [52]. To our knowledge, no previous work has implemented immersive VR allowing overground walking supported by passive walking braces. Its two‐phase design directly addresses one of the key barriers in assistive technology adoption: the transition from structured rehabilitation to real‐life application. Assessments of user experience and motion sickness will clarify both technical feasibility and the perceptual impact of VR‐based rehabilitation.

If successful, this approach could serve as the foundation for larger controlled studies. The ultimate goal is to integrate this system into routine clinical practice, supporting decision‐making in walking aid prescription and patient adaptation, and ultimately enhancing the autonomy and quality of life of people with MS. Moreover, optimizing the selection of orthoses could reduce the time and resources needed for clinical evaluations. Real‐time insights into fatigue and discomfort in walking may also help clinicians identify demanding scenarios associated with brace use and tailor interventions accordingly. In particular, markerless gait analysis will be compared with wearable sensors to further improve system usability and scalability. Preliminary ML pipelines will also be incorporated into the gait analysis as a proof‐of‐concept to evaluate the technical feasibility of processing inertial data for future automatic gait quality indexing. To the best of our knowledge, no previous study has proposed a technological clinical framework combining VR with passive walking braces, making this a novel and underexplored direction with important implications for both research and clinical practice.

Nevertheless, several limitations should be acknowledged. First, potential side effects of VR, such as cybersickness, may influence user experience. However, existing literature suggests that walking‐based navigation in VR significantly reduces discomfort compared to passive movement [53, 54]. Thus, minimal cybersickness or discomfort is expected in our protocol. Second, the simultaneous use of multiple devices, including the VR headset, VIVE trackers, mTest3 sensors, and the EmbracePlus wristband, may interfere with gait.

Beyond technical limitations, successful translation of VR and sensor‐based evaluations into routine clinical workflows requires careful consideration of the operational burden placed on both patients and clinical sites, as highlighted in recent frameworks for digital health technology deployment [55]. For patients, the simultaneous use of multiple wearables and VR headsets can induce physical or cognitive fatigue, particularly in individuals with MS who already experience disease‐related exhaustion. In our protocol, this is mitigated through short‐duration test protocols, structured rest intervals, and the availability of mixed‐reality modes to minimize cybersickness. For study sites and clinical staff, barriers include the time needed for multi‐device setup, sensor calibration, software management, and raw data integration. To minimize site burden and support scalability, future iterations of the VIRTUE framework will prioritize streamlining the sensor kit, such as evaluating whether video‐based markerless tracking can replace shoe‐worn IMUs and developing automated software pipelines to simplify data synchronization and reporting for clinicians. This user‐centered perspective will also extend to home‐based assessments, aiming to simplify setup requirements and minimize participant burden during ecological monitoring of daily activities. Ultimately, the present study is crucial for gathering direct feedback from both patients and clinicians about the usability and feasibility of the proposed instrumentation, laying the groundwork for optimizing the framework prior to its deployment in larger cohort studies.

Lastly, the small sample size of the study necessitates repeated testing across four experimental conditions (VR on/off and ExoBand on/off) to achieve meaningful results, instead of having two separate groups, one using VR and the other in the non‐VR condition. This procedure must be completed in a single session, as splitting it across 4 days (one for each condition) would create organizational challenges for participants, who may not be able to come to the hospital on four consecutive days, and would also lead to substantial time‐related costs for the hospital staff. To minimize fatigue‐related biases, rest breaks will be provided. Future studies with larger cohorts will allow for more robust statistical analyses and group comparisons. Additionally, the limited subject cohort poses an inherent risk of model overfitting when exploring ML pipelines. Although each participant contributes multiple gait repetitions, yielding numerous segmented gait cycles as individual analysis instances, these repeated observations are not statistically independent. In our protocol, the risk of overfitting and data leakage is constrained by utilizing LOSO‐CV, ensuring all gait cycles from a given participant are held out together during testing, alongside simple regularized models. Consequently, any preliminary ML performance metric must be interpreted strictly as an indicator of automated workflow feasibility rather than a generalizable predictive result.

In summary, this protocol investigates the feasibility of a novel, multidimensional approach to gait rehabilitation with assistive devices in people with MS. By combining VR, wearable sensors, and advanced data analysis, it offers a pathway to overcome the limitations of conventional clinical methods, moving toward more personalized, scalable, and clinically relevant strategies.

## Author Contributions

Kevin Marcaccini and Silvia Orlandi wrote the manuscript; Kevin Marcaccini, Alessandro Aleo, Stefania Pozzi, Giorgia Sgambato, Lorenzo Chiari, Maria Giovanna Verrengia, Domenica Chiara Milano, Serena Moscato, Sabato Mellone, Valerio Antonio Arcobelli, Loredana Sabattini, Silvia Orlandi designed the research; Loredana Sabattini, Kevin Marcaccini, Stefania Pozzi, Giorgia Sgambato performed the research.

## Funding

The publication of this article was supported by the “Ricerca Corrente” funding from the Italian Ministry of Health and funded by the Fondazione del Monte di Bologna e Ravenna (grant number 2023.0491).

## Ethics Statement

The study has received approval from the local Ethics Committee (approval no. 29‐2025‐DISP‐AUSLBO). Results will be published in international peer‐reviewed journals, and summaries will be provided to the patients of the study.

## Conflicts of Interest

The authors declare no conflicts of interest.

## Supporting information


**Data S1:** Supporting Information.

## Data Availability

The authors have nothing to report.
